# Do our newly graduated medical doctors have adequate knowledge about neonatal resuscitation?

**DOI:** 10.1590/S1516-31802007000300010

**Published:** 2007-05-03

**Authors:** Ana Paula de Carvalho Panzeri Carlotti, Maria Lúcia Silveira Ferlin, Francisco Eulógio Martinez

**Keywords:** Newborn, Resuscitation, Practice guideline, Teaching, Internship and residency, Recém-nascido, Ressuscitação, Diretrizes para a prática clínica, Ensino, Internato e residência

## Abstract

**CONTEXT AND OBJECTIVE:**

Neonatal resuscitation should be part of medical school curriculums. We aimed to evaluate medical school graduates’ knowledge of neonatal resuscitation.

**DESIGN AND SETTING:**

Cross-sectional study on the performance of candidates sitting a medical residency exam at Hospital das Clínicas, Faculdade de Medicina de Ribeirão Preto, Universidade de São Paulo, in 2004.

**METHODS:**

There were two questions on neonatal resuscitation. One question in the theory test aimed at evaluating basic knowledge on the initial approach towards newly born infants. The question in the practical exam was designed to evaluate the candidate's ability to perform the initial steps of resuscitation and to establish bag-mask ventilation.

**RESULTS:**

Out of 642 candidates from 74 medical schools, 151 (23.5%) answered the theory question correctly. Significantly more physicians from public medical schools in the State of São Paulo answered correctly than did those from other schools in Brazil (52.5% versus 9.2%; p < 0.05). A total of 436 candidates did the practical exam. The grades among graduates from medical schools belonging to the State of São Paulo were significantly higher than among those from other schools (5.9 ± 2.6 versus 4.1 ± 2.1; p < 0.001). The grades for the practical question among candidates who had answered the theory question correctly were significantly higher than those obtained by candidates who had given wrong answers (p < 0.05).

**CONCLUSION:**

Medical school graduates’ knowledge of neonate resuscitation in the delivery room is quite precarious. Emphasis on neonatal resuscitation training is urgently needed in medical schools.

## RESUMO

**CONTEXTO E OBJETIVO:**

A reanimação neonatal deveria fazer parte do curriculum médico. Nosso objetivo foi avaliar os conhecimentos básicos sobre reanimação neonatal de egressos de faculdades de medicina.

**TIPO DE ESTUDO E LOCAL:**

Estudo transversal do desempenho dos candidatos que participaram do concurso para residência médica no Hospital das Clínicas da Faculdade de Medicina de Ribeirão Preto da Universidade de São Paulo em 2004.

**MÉTODOS:**

O exame tinha duas questões: uma teórica, com o objetivo de avaliar o conhecimento dos primeiros passos da recepção e uma prova prática, que pretendia avaliar a habilidade do candidato em executar os primeiros passos da ressuscitação e a ventilação com bolsa e máscara.

**RESULTADOS:**

Dos 642 candidatos provenientes de 74 faculdades, 151 (23,5%) acertaram a questão teórica. A taxa de acerto foi significativamente maior entre os egressos das escolas médicas públicas do Estado de São Paulo quando comparadas com as demais escolas do Brasil (52,5% *versus* 9,2%, p < 0,05). Realizaram a prova prática 436 candidatos. As notas dos egressos das escolas estaduais de São Paulo foram significativamente maiores do que as notas dos egressos das demais faculdades (5,9 ± 2,6 *versus* 4,1 ± 2,1, p < 0,001). As notas obtidas no exame prático pelos alunos que acertaram a questão teórica foram significativamente maiores do que as notas obtidas pelos alunos que erraram (p < 0,05).

**CONCLUSÃO:**

O conhecimento dos egressos das escolas médicas sobre o atendimento ao recém-nascido na sala de parto é muito precário, existindo, portanto, a premente necessidade de que seja dada maior ênfase ao ensino da reanimação neonatal durante a graduação em medicina.

## INTRODUCTION

Care for newborns in the delivery room used to be traditionally based on the Apgar score.^1–3^ However, after the publication of a neonatal resuscitation manual by the American Academy of Pediatrics and the American Association of Cardiology in 1987, a new paradigm was created for neonatal resuscitation. The guidelines recommended by the manual started to be gradually incorporated by different countries, including Brazil, and underwent small revisions and modifications over the years.^4–8^

At the center of these guidelines is the concept that systematic, rapid and effective care for newborns in the delivery room is fundamental for preventing injuries due to asphyxia, which may lead to neurological sequelae or even death. This premise has been thoroughly evaluated and the program has been shown to have significant consequences for infants.^9–11^ Major improvements in Apgar scores and outcomes for neonates, and significant reduction in mortality of term and premature babies have been demonstrated following the implementation of neonatal resuscitation training programs.^11–14^

Currently, there is a consensus that teaching of neonatal resuscitation should be part of every institution's curriculum and that all medical schools should have a team responsible for this task. This training should be a fundamental requirement for medical and nursing education, and competence should be expected from students and graduates.^14–18^ However, these requirements have not been accomplished. A study on neonatal resuscitation teaching at 36 public maternity hospitals in Brazilian state capitals showed that in 15% of the institutions that had undergraduate doctors and in 88% of the institutions that had undergraduate nurses who had clinical activities in the delivery room, these students cared for neonates without any specific training.^19^ A recent survey carried out in Australia showed that approximately one-third of health personnel involved in deliveries in rural and urban non-tertiary units, where three-quarters of all births take place, had been inadequately trained in neonatal resuscitation.^20^ In this context, evaluation of neonatal resuscitation teaching is essential, to establish what need there is for educational interventions, so as to improve the quality of care for newborns in the delivery room.

## OBJECTIVE

In the present study, the authors aimed to evaluate the basic knowledge of neonatal resuscitation among newly graduated medical doctors.

## MATERIAL AND METHODS

This was a cross-sectional study on the performance of candidates who sat an exam for medical residency at Hospital das Clínicas, Faculdade de Medicina de Ribeirão Preto, Universidade de São Paulo (HC/FMRP/USP), in 2004. Two questions (one on theory and the other on practice) aimed at evaluating candidates’ knowledge of resuscitation of newly born infants in the delivery room were included in the exams for selecting medical residents for HC/FMRP/USP. The first question formed part of the theory test and it aimed to evaluate basic knowledge on the initial approach of newly born infants. Annex 1 presents the theory question on neonatal resuscitation.

The candidates selected for the second phase of the exam did a practical question on neonatal resuscitation that was designed to evaluate the candidate's ability to perform the initial steps of resuscitation and to establish bag-mask ventilation. The task was set up as if the candidate were in a room at the obstetrics center where a baby was going to be born. The environment was properly organized as a delivery room with a radiant warmer, an oxygen tube, suction catheters, a self-inflating bag with an oxygen reservoir, face masks of premature and newborn sizes, a laryngoscope with straight blades (Nos. 0, 1 and 2) and a curved blade (No. 1), and tracheal tubes of 2.5, 3.0 and 3.5 mm. A sound system and a manikin were also in the room but not visible to the candidate. The following information was provided on a poster:

*“A pregnant woman in expulsive labor arrives without having received prenatal care and an apparently term baby is born. The newly born infant is cyanotic and presents the heart rate that you are hearing. Deal with this manikin as if it were the baby”.*

While the candidate read the poster, the examiner switched on the sound system, which presented a recording of a rhythmic heartbeat of 50 beats per minute. As soon as the candidate finished reading the poster, a wrapped manikin was delivered to him/her. The candidate was expected to perform the following actions: 1) Place the manikin under the radiant warmer; 2) Position the head; 3) Suction the mouth; 4) Suction the nostrils; 5) Dry; 6) Remove the damp cloth; and 7) Assess. Since the heart rate continued at 50 beats per minute, the candidate was expected to continue to intervene with the following actions: 8) Apply to the face a mask of appropriate size attached to a self-inflating bag with oxygen reservoir; 9) Perform bag-mask ventilation at adequate rate; and 10) Obtain adequate chest expansion with ventilation. As the candidate performed the actions, the examiner recorded each of them in the order in which they were performed, on a checklist. The time available to each candidate was 3.5 minutes. The actions were divided into two stages: the first comprised the initial steps of resuscitation, with a maximum score of 6, and the second concerned the establishment of bag-mask ventilation, with a maximum score of 4.

In order to score 6 in the first stage, the candidate was supposed to perform all the actions in the right sequence. When all the actions were performed, but in a wrong sequence, the candidate scored 4. One point was lost for each action that was not performed, but when suctioning of the airways was not done the candidate scored zero. In the second stage, the candidates scored 4 when they applied the appropriate facemask attached to a self-inflating bag with oxygen reservoir, ventilated at the correct rate, and obtained adequate chest expansion. For each action that was not performed or that was performed in an inadequate manner, one point was subtracted. The use of an oxygen reservoir or the attachment of the oxygen tube to the bag was not considered for grading purposes because of the possible debate regarding the use of oxygen for resuscitating depressed neonates.^21^ Candidates who tried to intubate before establishing bag-mask ventilation scored zero in the second stage. Those who performed chest compressions before the first steps or before bag-mask ventilation scored zero for the whole question.

The data were analyzed by analysis of variance (ANOVA), with Tukey multiple *post hoc* comparisons, using the Statistical Package for the Social Sciences (SPSS) 12.0 software, with the level of significance set at p < 0.05. The data were expressed as mean ± standard deviation (SD) and 95% confidence interval (CI).

## RESULTS

A total of 642 candidates sat the medical residency exam at HC/FMRP/USP in 2004; 80.9% were newly graduated medical doctors, 12.2% had graduated in the preceding year, 4.4% two years before, and 2.5% had graduated more than two years earlier. Two candidates were foreigners (from Peru and Cuba). Table 1 shows the distribution of Brazilian candidates according to the type of medical school of origin and the Federal State where it is located. The candidates from institutions in the State of São Paulo were from 6 state schools, 2 municipal schools and 10 private schools. There were no candidates from the Federal University (Universidade Federal de São Paulo; Unifesp). The candidates from other States in Brazil came from 5 state schools, 27 federal schools and 24 private schools.

**Table 1.** Distribution of Brazilian candidates who applied to take medical residency exams at Hospital das Clínicas, Faculdade de Medicina de Ribeirão Preto, Universidade de São Paulo (HC/FMRP/USP), in 2004, according to the type of medical school of origin and the Federal State where this institution is located

	Type of medical school	Number of schools	Mean number of candidates per school	Total number of candidates
São Paulo	State	6	35.83	215
	Municipal	2	7.50	15
	Private	10	10.90	109
Other States	State	5	5.60	28
	Federal	27	6.33	171
	Private	24	4.25	102
**Total**		**74**	**8.65**	**640**

The theory question on neonatal resuscitation was analyzed for level of difficulty and power of discrimination. According to the method of Heraldo Maretim Vianna,^22^ the theoretical question had a discrimination index of 40% and a difficulty index of 76.46%. Table 2 shows the numbers of answers given for each of the alternatives of the theory question. Considering “A” as correct, we observed that 151 candidates (23.5%) responded correctly to the question. The numbers and percentages of correct answers for the theory question according to the type of medical school of origin are listed in Table 3. It should be pointed out that data are only presented for 640 candidates, because the candidates from foreign institutions were excluded. It can be seen that the results obtained by candidates from medical schools belonging to the State of São Paulo were significantly different from those obtained by candidates from the other schools (p < 0.05).

**Table 2.** Responses to the theory question on neonatal resuscitation, according to the alternatives

	**Alternatives**				
	A	B	C	D	E
Number of responses	151	112	158	195	26
Percentage	23.5%	17.5%	24.6%	30.4%	4.0%

**Table 3.** Responses to the theory question on neonatal resuscitation, according to the type of medical school of origin

	Type of medical school	Correct response (n)	(%)	Incorrect response (n)	(%)	Total number of candidates
São Paulo	State*	113	52.6	102	47.4	215
	Municipal	4	26.7	11	73.3	15
	Private	7	6.4	102	93.6	109
Other States	State	0	0	28	100	28
	Federal	17	9.9	154	90.1	171
	Private	11	10.8	91	89.2	102
**Total**		**152**	**23.75**	**488**	**76.25**	**640**

* *Analysis of variance, ANOVA; p < 0.05 (Tukey post hoc multiple comparison).*

A total of 436 candidates went on to do the practical exam. Table 4 shows the distribution of the grades in the practical question on neonatal resuscitation according to the stage of the question and the type of medical school of origin. In the first stage of the question, the grades obtained by candidates from medical schools belonging to the State of São Paulo were higher than those obtained by candidates from other schools. In the second stage, the grades obtained by candidates from São Paulo state schools were similar to those obtained by candidates from other state schools but significantly different from other schools in Brazil. The total grades obtained by candidates from São Paulo state schools were similar to those obtained by candidates from other state schools but significantly different from other schools in Brazil. The grades of graduates from São Paulo state schools were significantly higher than those from all other schools (5.9 ± 2.6 versus 4.1 ± 2.1; 95% CI: 5.5–6.3 versus 3.9–4.4; p < 0.001). When the candidate's choice of specialization was evaluated for possible impact on the grades, no difference was detected among the candidates for the various specialties. The grades in the practical question on neonatal resuscitation obtained by candidates who applied to do residency in Pediatrics were similar to those obtained by candidates for other specialties (Table 5). In addition, there was a marked consistency in the replies to the theoretical and practical questions on neonatal resuscitation, since grades obtained in both stages of the practical question by candidates who answered the theory question correctly were significantly higher than those obtained by candidates who gave an incorrect reply to the theory question (Table 6).

**Table 4.** Distribution of grades in the practical question on neonatal resuscitation, according to the stage of the question and the type of medical school of origin

	Type of medical school	n	Stage 1	Stage 2	Total
São Paulo	State	167	3.53 ± 1.83*	2.38 ± 1.38†	5.92 ± 2.64†
	Municipal	69	2.70 ± 1.62	1.26 ± 1.34	3.96 ± 2.14
	Private	11	1.91 ± 1.22	1.09 ± 0.94	3.00 ± 1.84
Other States	State	16	2.13 ± 1.78	2.81 ± 1.17‡	4.94 ± 2.11
	Federal	119	2.52 ± 1.63	1.68 ± 1.28	4.20 ± 2.27
	Private	54	2.70 ± 1.41	1.48 ± 1.26	4.19 ± 1.97
**Total**		**436**	**2.93 ± 1.74**	**1.89 ± 1.39**	**4.81 ± 2.51**

*Analysis of variance, ANOVA; p < 0.05 (Tukey* post hoc *multiple comparison);*

* *significantly different from all the others;*

† *São Paulo state schools similar to other state schools and significantly different from all others;*

‡ *state schools in other States similar to São Paulo state schools and significantly different from all others.*

**Table 5.** Grades obtained in the practical question on neonatal resuscitation, according to the candidate’s choice of specialty

Practical question	Choice	n	Mean	Standard deviation	p
Stage 1	Pediatrics	71	2.84	1.57	0.66
	Others	365	2.94	1.77	
Stage 2	Pediatrics	71	1.90	1.33	
	Others	365	1.88	1.40	0.91
**Total**	**Pediatrics**	**71**	**4.74**	**2.26**	
	**Others**	**365**	**4.82**	**2.55**	**0.80**

**Table 6.** Grades obtained in the practical question on neonatal resuscitation, according to the results from the theory question on neonatal resuscitation

Practical question	Theory question	n	Mean	Standard deviation	95% confidence interval	p
Stage 1	Correct responses	132	3.86	1.81	3.55	4.18	**< 0.001**
	Incorrect responses	304	2.52	1.55	2.35	2.69	
Stage 2	Correct responses	132	2.46	1.35	2.23	2.69	
	Incorrect responses	304	1.63	1.34	1.48	1.79	**< 0.001**
**Total**	**Correct responses**	**132**	**6.33**	**2.58**	**5.89**	**6.78**	
	**Incorrect responses**	**304**	**4.15**	**2.17**	**3.90**	**4.40**	**< 0.001**

## DISCUSSION

After the enormous progress in the care provided for neonates in the delivery room that was triggered by the score developed by Virginia Apgar,^1^ more than 30 years elapsed without any further significant development in this field. Following the institution of the Neonatal Resuscitation Program, a very well structured course proposed by the American Academy of Pediatrics and the American Association of Cardiology, which was subsequently adapted by Unifesp and the Brazilian Society of Pediatrics (Sociedade Brasileira de Pediatria, SBP), the new guidelines for caring for neonates in the delivery room have been widely disseminated among pediatricians.^4–8^

Considering that the transition from intrauterine to extrauterine life is probably the single most dangerous event faced by a human being in his or her lifetime, it is crucial that not only pediatricians but also all medical doctors have the basic knowledge needed to help neonates during this period. Since junior medical staff are frequently on the frontline of neonatal resuscitation, adequate neonatal resuscitation training is fundamental to the medical curriculum.^14–18^ The medical residency exam represents an excellent opportunity to assess the knowledge acquired during graduation, not only because most candidates are newly graduated medical doctors, but also because of the possibility of evaluating physicians from different regions of the country.

In 2004, 642 candidates applied to take the medical residency exam at HC/FMRP/USP. This number was much lower than what had traditionally been seen, possibly due to the fact that this was the first occasion on which a practical exam was undertaken, requiring the candidate to take exams on two consecutive weekends: one for the theory test and the other for the practical exam. This requirement meant that the candidates who had passed the theoretical phase were unable to take exams at other locations on the following weekend. However, it was an interesting sample for analysis since about 50% of the candidates were from the State of São Paulo and 50% were from other Federal States, and most of them were newly graduated medical doctors.

The objective of the theory test was to assess the candidates’ knowledge about basic care to be provided at birth. Initially based on a consensus among specialists, the neonatal resuscitation guidelines have become more uniform since the implementation of the Neonatal Resuscitation Program. The revisions of the program that followed the first proposal have definitely resulted in significant refinement of the guidelines. One interesting point in these revisions was the understanding that not all newborns need to be actively manipulated at birth. Most term infants who are born in good condition can frequently be placed in direct skin-to-skin contact with their mothers before being subjected to uncomfortable stimuli such as aspiration of the upper airways and routine care like intramuscular injection of vitamin K, vaccination against hepatitis B and ocular application of silver nitrate.

The fact that only 23.5% of the candidates correctly answered the theory question on neonatal resuscitation strongly indicates that neonatal resuscitation training has not been properly emphasized in medical schools, as already assumed by Almeida et al.^19^ It is interesting to point out that the highest proportion of candidates (30.4%) considered alternative D to be correct (Annex 1). In this alternative, the first steps of resuscitation of a newly born infant are correct, as recommended by the guidelines.^6^ However, this alternative does not take into account one fundamental matter, i.e., assessment of the resuscitation requirement. Separating a newly born infant from his/her mother when there is no need for this is the opposite of what is recommended by good practice at a child-friendly hospital. In alternative D, the newly born infant is taken away from the mother and returns to her only after routine procedures. This is a hospital practice that, unfortunately, still seems to be ingrained in most institutions, considering that about one third of the candidates thought that this was the appropriate response.

The second most frequently chosen alternative in the theory question was C, which contained the earlier recommendations on neonatal resuscitation.^4^ This indicates that, in some medical schools, the information provided to the students may be out-of-date. It should be pointed out that variability in delivery room resuscitation practices has also been detected in the United States.^23^

Analysis of the type of medical school also provided important information. The schools were divided into two groups: São Paulo state schools and schools from other states. This separation allowed us to practically divide the study population down the middle. The percentage of correct responses to the theory question about neonatal resuscitation was much higher among candidates from São Paulo state schools than among those from other schools, thus indicating that the former had access to more up-to-date information. This picture was virtually repeated among the candidates who took the practical exam. Considering 50% as the cutoff point, i.e. a score of 3.0 in the first stage and 2.0 in the second, it can be seen that only the candidates from São Paulo state schools reached this level of performance. The remaining state schools were able to exceed 50% only in the second stage of the practical question and most candidates from other Brazilian medical schools were well below this limit. Furthermore, despite the discrimination index of 40% presented by the theory question on neonatal resuscitation, consistency between the responses to the theory and the practical questions was well demonstrated. This is a very important matter that attests to the reliability of the measurement made. Although there is the possibility of random guessing of correct responses in the theory test, this is completely ruled out in the practical exam.

The choice of specialty did not influence the candidates’ performance. Although we aimed to evaluate the performance of general physicians before specialization, there is no doubt that every medical doctor should have adequate basic knowledge of neonatal resuscitation, as already recommended.^14–18^ Considering the results as a whole, this study has clearly demonstrated that the performance of candidates from all the schools was quite inadequate, since only 15 of them (3.4%) obtained the highest score in the practical question on neonatal resuscitation (all of them had answered the theory question correctly). In other words, what was expected as a rule represented an exception.

## CONCLUSIONS

Newly graduated medical doctors’ knowledge regarding care for neonates in the delivery room is quite precarious. Greater emphasis on neonatal resuscitation training is urgently needed during the medical course.

## Annex 1. Theory question on neonatal ressuscitation in the exam for medical residency at Hospital das Clínicas, Faculdade de Medicina de Ribeirão Preto, Universidade de São Paulo in 2004

**Table:**

You are called to care for a newly born infant. Obstetrics data show that the mother is a 25-year-old primigravida who attended seven prenatal visits without any problems and who is now in the 40th week of pregnancy according to the date of her last menstruation. Labor is progressing with no alterations and a neonate is born by vaginal delivery, with the need for episiotomy. What is the appropriate approach?
a) As soon as the baby is born, determine whether there is meconium, prematurity, atony, cyanosis or respiratory difficulty. If none of these are present, place the neonate with the mother, with no other manipulation.
b) Place the baby under a radiant heat source and evaluate heart rate, respiratory rate and skin color. If everything is OK, perform a physical examination and take the baby to the mother.
c) Place the baby under a radiant heat source. Dry, remove the damp cloths, suction the nostrils and then the mouth, and position the baby. If everything is OK, take the baby to the mother.
d) Place the baby under a radiant heat source. Position, suction the mouth and then the nostrils, dry, remove the damp cloths and position the baby. Perform a complete physical examination. If everything is OK, send the neonate to the nursery for a bath, and apply vitamin K intramuscularly and 1% silver nitrate to the eyes. When the mother is feeling well, take the baby to her.
e) Place the baby under a radiant heat source. Apply the stethoscope to the precordium to check the heart rate and determine the Apgar score at one minute. If the Apgar score is higher than 7, suction the nostrils and mouth, and perform a physical examination. If everything is OK, send the neonate to the nursery for a bath, and apply vitamin K intramuscularly and 1% silver nitrate to the eyes. When the mother is feeling well, take the baby to her.
Alternative A was considered to be correct.
